# Supplementary material for: Coordination of virulence factors and lifestyle transition in Pseudomonas aeruginosa through single-cell analysis
Source: Commun Biol. 2025 Aug 16;8:1236. doi: 10.1038/s42003-025-08693-6 (PMC12357884; doi:10.1038/s42003-025-08693-6)
Supplement: Supplementary file 2 — Supplementary Information [file 42003_2025_8693_MOESM2_ESM.pdf]

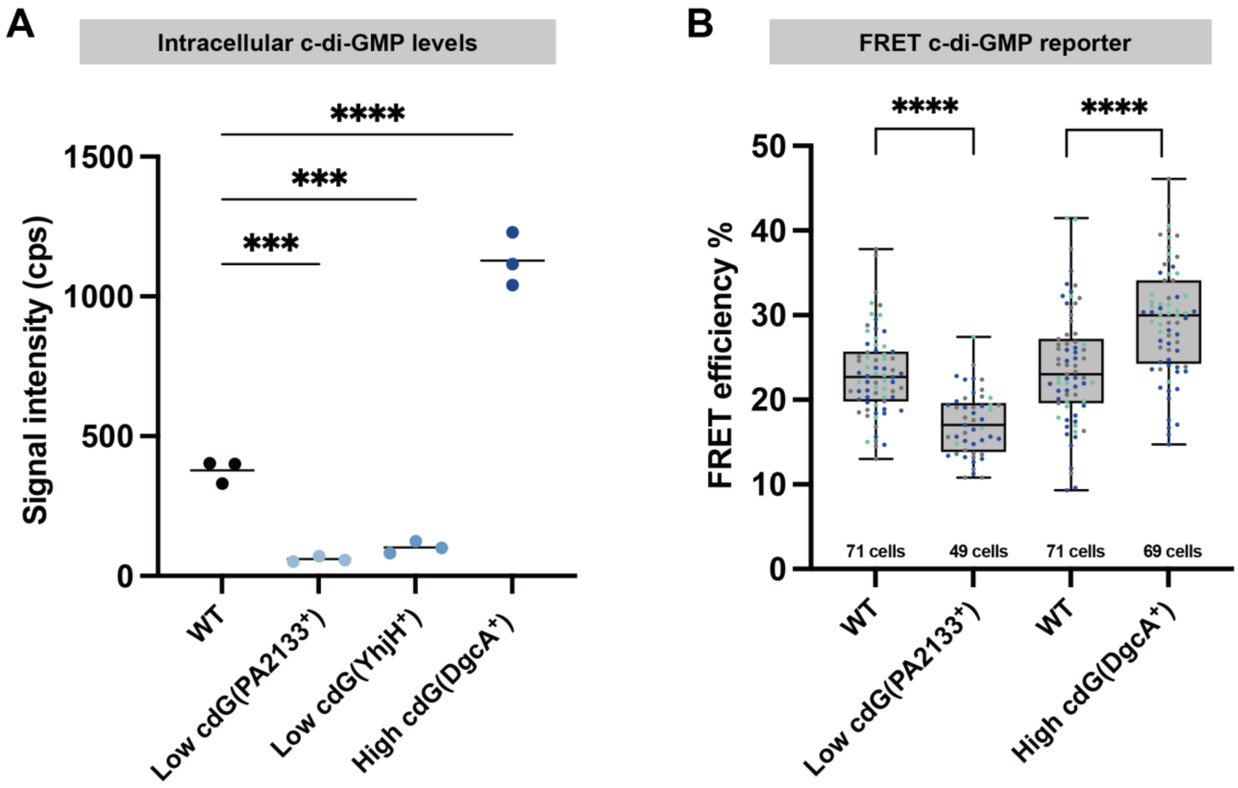

Suppl. Fig. 1 - Validation of the functionality of c-di-GMP metabolic enzymes used in this work using different c-di-GMP detection methods.

A) Intracellular c-di-GMP abundance in bacterial strains used in this study. Data shows integrated peak area signal intensity of targeted LC-MS/MS measurements, see Methods for detailed calculations. Biological replicates, n=3. Lines denote mean intensity. Statistical analysis was done via ANOVA with multiple comparison to WT, ***, $p< 0.001$; ****, $p< 0.0001$. **B**) Validation of the FRET biosensor in *P. aeruginosa* strains in different c-di-GMP backgrounds. Data is shown as corrected FRET efficiency, see Methods for detailed calculations. WT (vector control in first box) and PA2133^{+} carried pUCP20-based vectors, and WT (vector control in third box) and DgcA^{+} carried pMMB67EH-based vectors. Each data point represents measurement of a single cell. Experiments were repeated on multiple dates, with each color set representing data points from one day to highlight reproducibility. Highest and lowest end of each bar represent maximum and minimum value, respectively; boxes indicate median and 25th-75th percentile. Total number of cells for each group is indicated at the bottom. Statistical analysis was done via Student's *t* test, ****, $p<0.0001$.

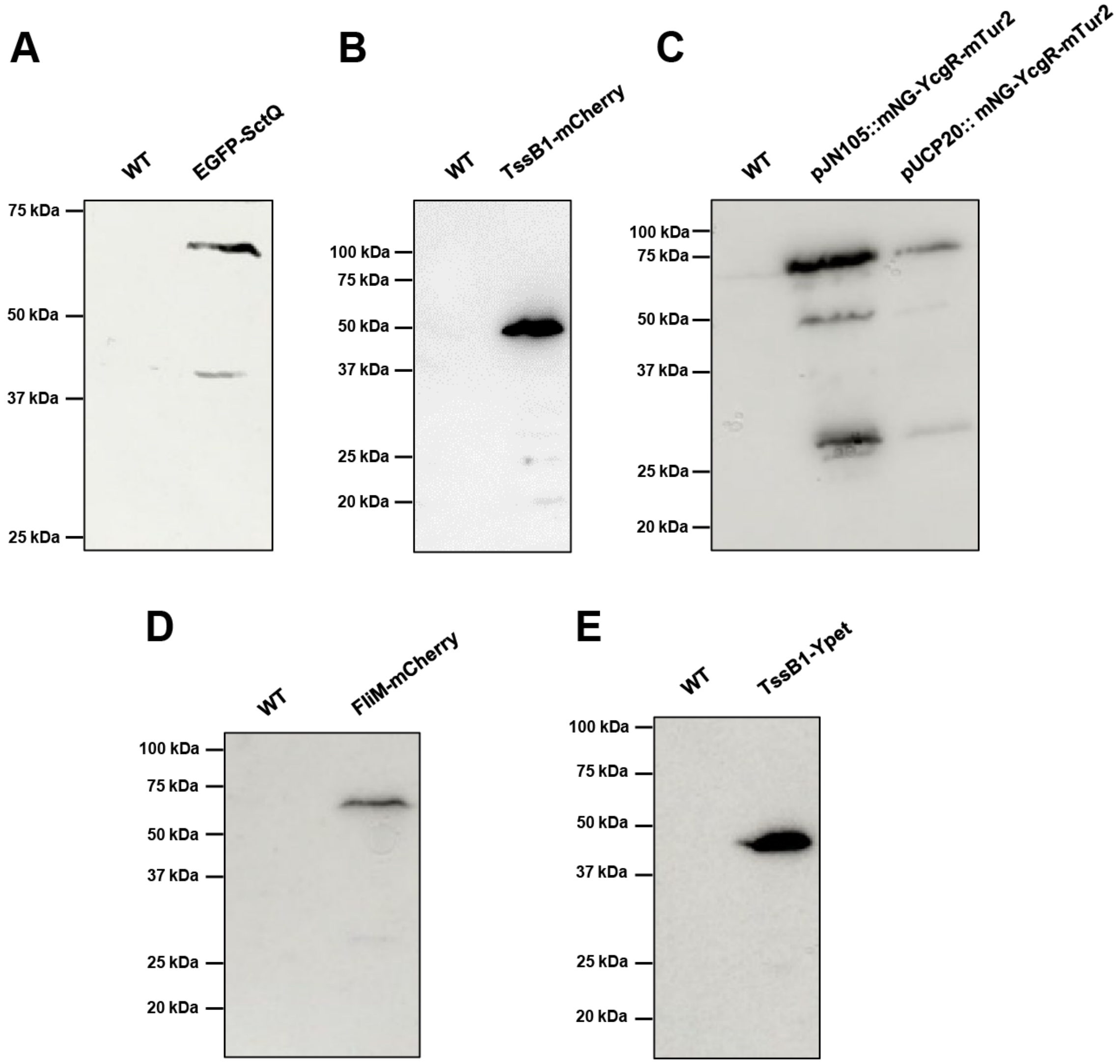

Suppl. Fig. 2 - Validation of expression and stability of fusion proteins used in this study.

Western blots using antibodies directed against GFP (A, C, E) or mCherry (B, D) to detect indicated fusion protein in total cell lysates. Except for C), all proteins are expressed as genomic fusions. Left, protein size standards. Expected sizes: EGFP-SctQ, 60 kDa; TssB1-mCherry, 48 kDa; mNeonGreen-YcgR-mTurquoise2 (mNG-YcgR-mTur2), 79 kDa; FliM-mCherry, 63 kDa; TssB1-Ypet, 47 kDa.

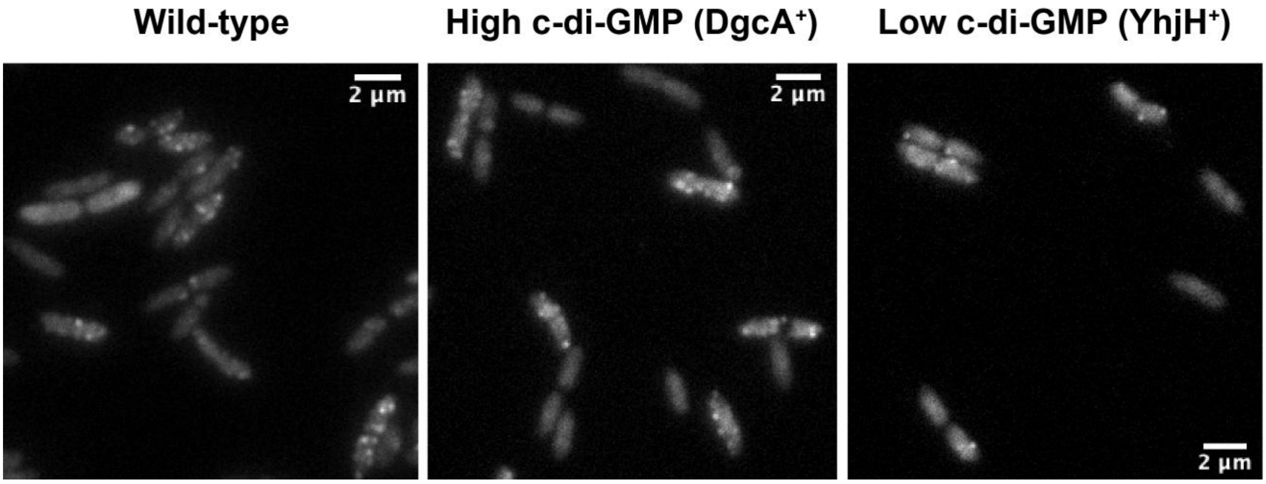

Suppl. Fig. 3 – Representative micrographs for the determination of the number of T3SS per bacterium.

Representative green channel micrographs of *P. aeruginosa* EGFP-SctQ, from which the number of T3SS of bacterium in the indicated strains was determined in Fig. 1B.

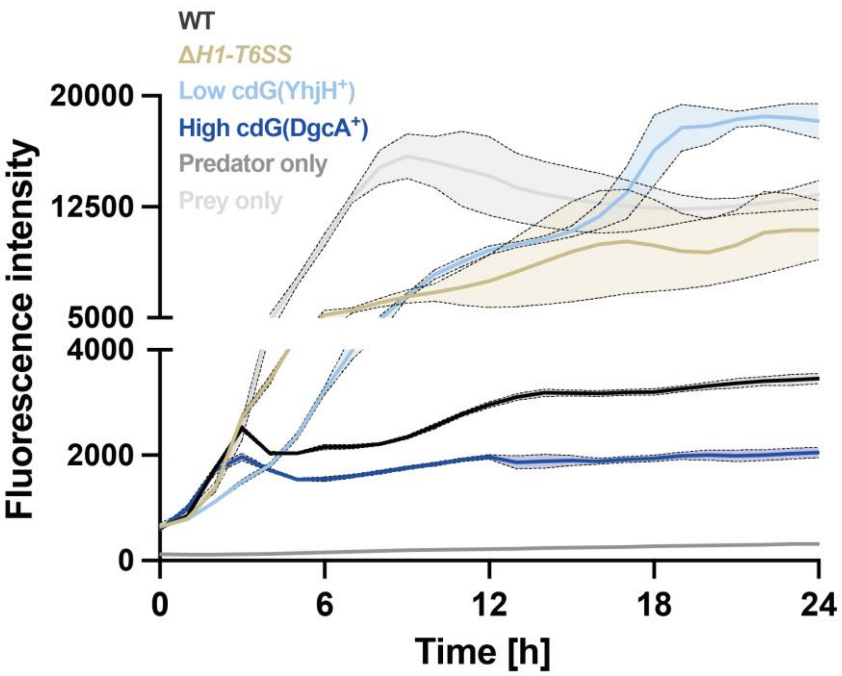

Suppl. Fig. 4 – Influence of c-di-GMP on H1-T6SS killing efficiency over time.

Data is shown as development of prey fluorescence over 24 hours from the initial mixture of predator and prey cells. Indicated strains were used as predators, while a YFP-labeled H1-T6SS effector-immunity pair deletion strain (Δ*tse6tsi6*) was used as prey. Fluorescence was read on plate reader with background subtracted. Biological replicates, n=3, lines and areas denote average values and standard deviation.

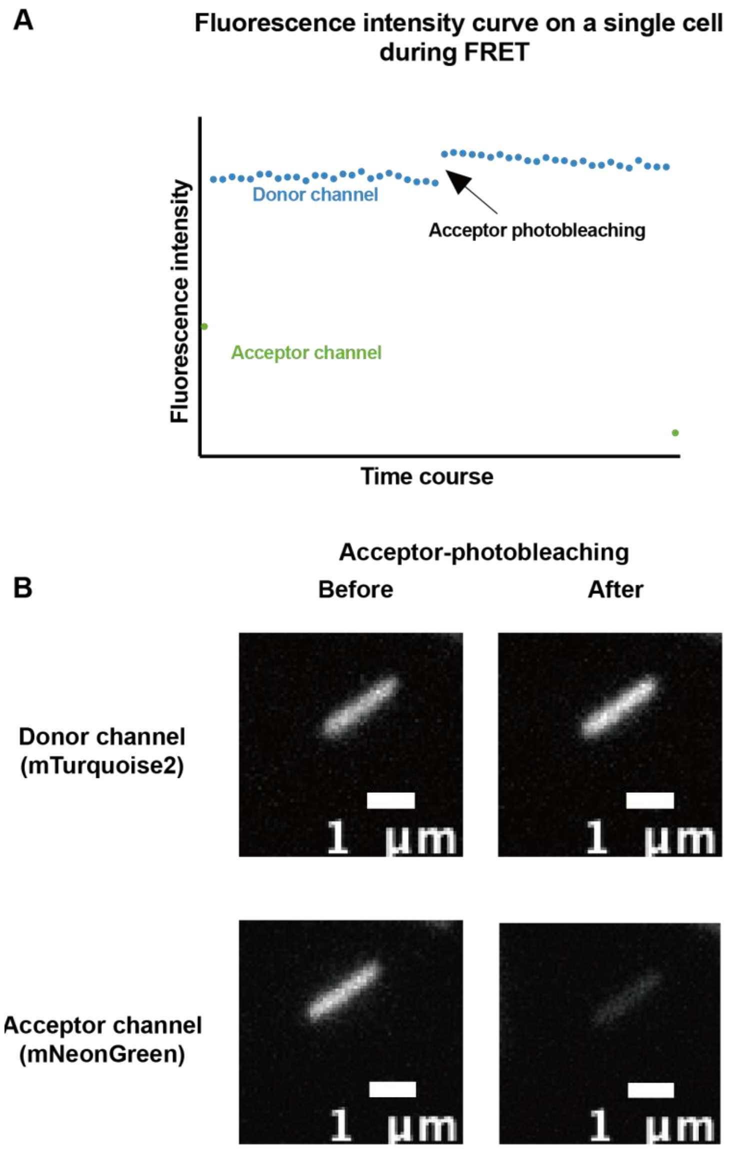

Suppl. Fig. 5 – Fluorescence intensity changes in the acceptor-photobleaching FRET.

A) Representative fluorescence intensity curve acquired on a single cell during acceptor-photobleaching FRET. Green data points indicate fluorescence acquired in the acceptor channel at the beginning and end of the experiment; blue data points indicate fluorescence acquired in the donor channel over time. Acceptor photobleaching performed at the time point indicated. **B**) Representative microscopy images from acceptor photobleaching FRET conducted in WT strain. Donor fluorescence increases, while acceptor fluorescence decreases after the acceptor photobleaching event.

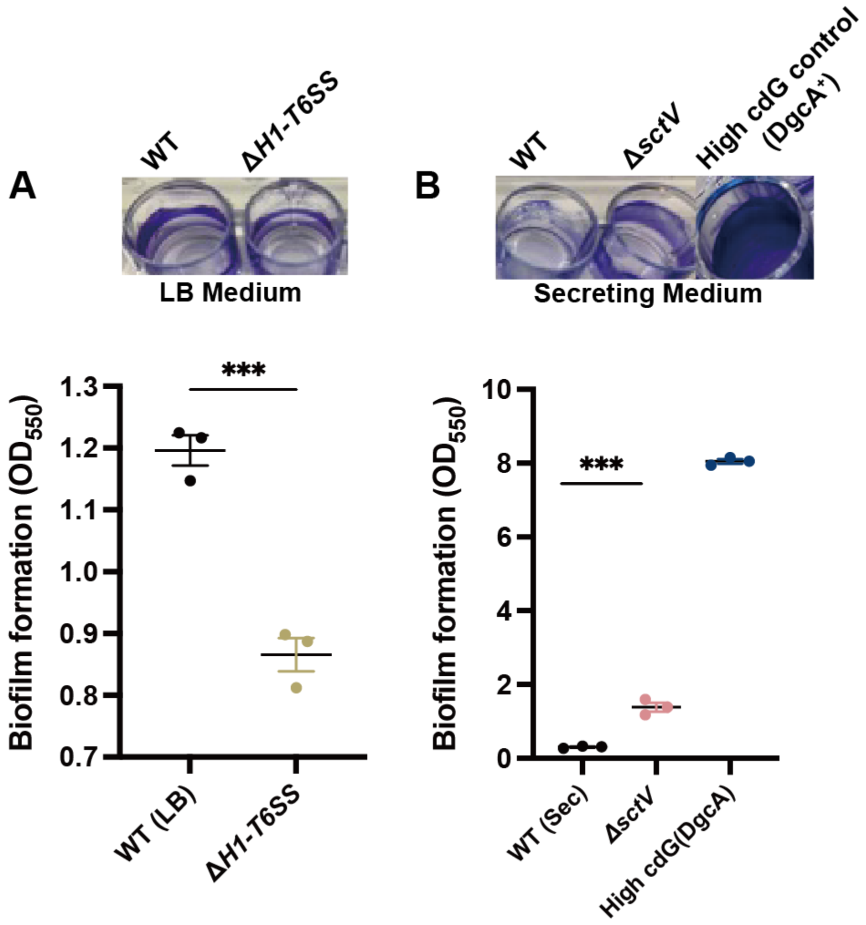

Suppl. Fig. 6 - T3SS presence decreases biofilm formation, while H1-T6SS increases biofilm formation.

A) ΔH1-T6SS and respective wild-type control were cultured in LB medium and biofilm formation was quantified by crystal violet staining and measurement of the optical density at 550 nm (OD_{550}). Top, representative pictures of biofilm staining for each strain tested. Lines denote mean value. Biological replicates, n=3; ***, $p< 0.001$. **B**) Wild-type control, high c-di-GMP (DgcA) control and the T3SS-negative Δ*sctV* were cultured in T3SS secreting medium and biofilm formation was quantified by crystal violet staining and measurement of the optical density at 550 nm (OD_{550}). Top, representative pictures of biofilm staining for each strain tested. Lines denote mean value. Biological replicates, n=3. Statistical analysis was done via ANOVA test, ***, $p< 0.001$

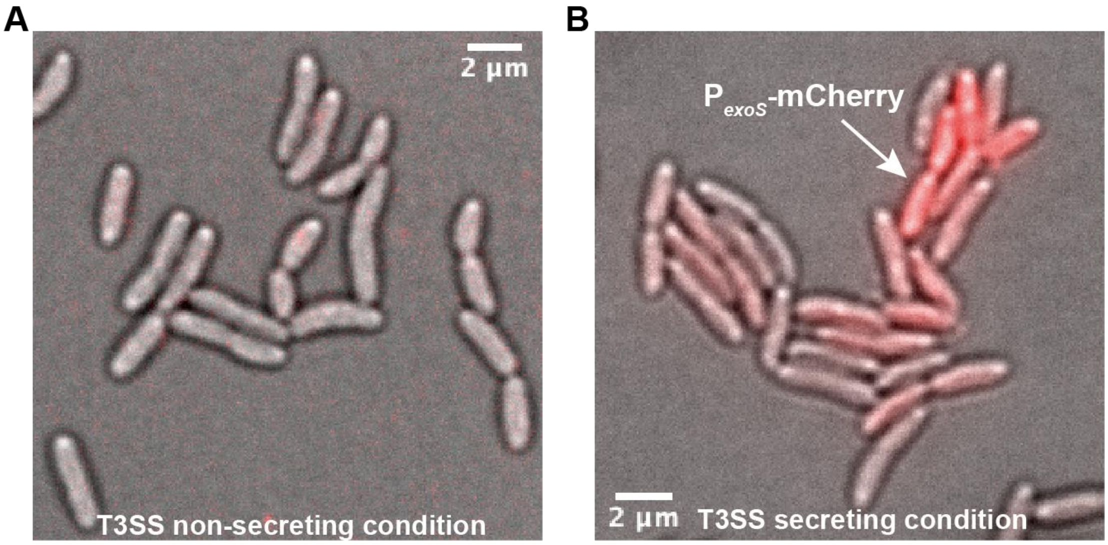

Suppl. Fig. 7 – The T3SS P$_{exoS}$-mCherry reporter is activated in T3SS secreting conditions.

Representative microscopy pictures with merged brightfield and red fluorescence channels of strains carrying the P$_{exoS}$-mCherry reporter cultivated in T3SS non-secreting conditions (**A**) or secreting conditions (**B**). Arrow indicates cells with intense reporter activity.

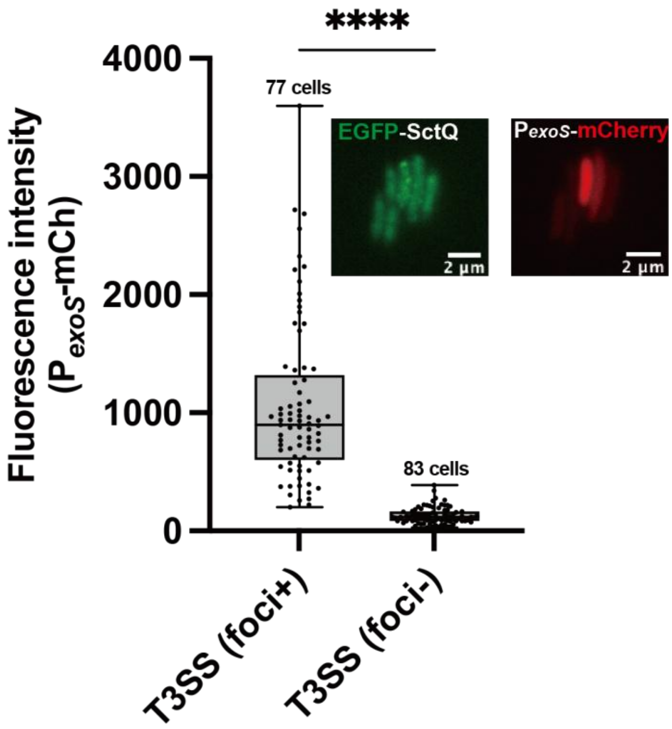

Suppl. Fig. 8 – P$_{exoS}$-mCherry T3SS reporter activity aligns well with T3SS assembly.

Microscopy analysis of background-corrected P$_{exoS}$-mCherry reporter fluorescence intensity in cells with or without assembled T3SS, as indicated by presence of EGFP-SctQ foci. Each data point represents the reporter intensity of one cell. Samples were taken from 8 different fields in 3 different experiments. Number of analyzed bacteria indicated at top. Highest and lowest end of each bar represent maximum and minimum value, respectively; boxes indicate median and 25th-75th percentile. ****, $p < 0.0001$. Microscopy pictures of same field of view showing EGFP-SctQ and P$_{exoS}$-mCherry in individual channels, as indicated.

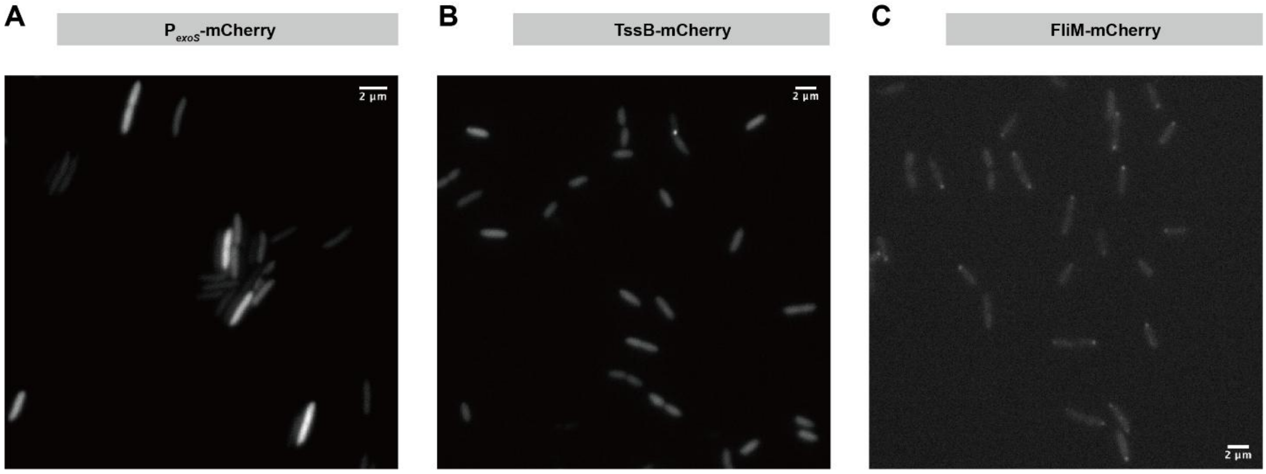

Suppl. Fig. 9 – Larger fields of view of the micrographs used for the analysis of the co-occurrence of T3SS, H1-T6SS, and FliM-mCherry.

Representative red channel micrographs of *P. aeruginosa* expressing the indicated proteins, from which the activity of the T3SS and the presence of the H1-T6SS or flagellum was determined in Fig. 3 and 5A.

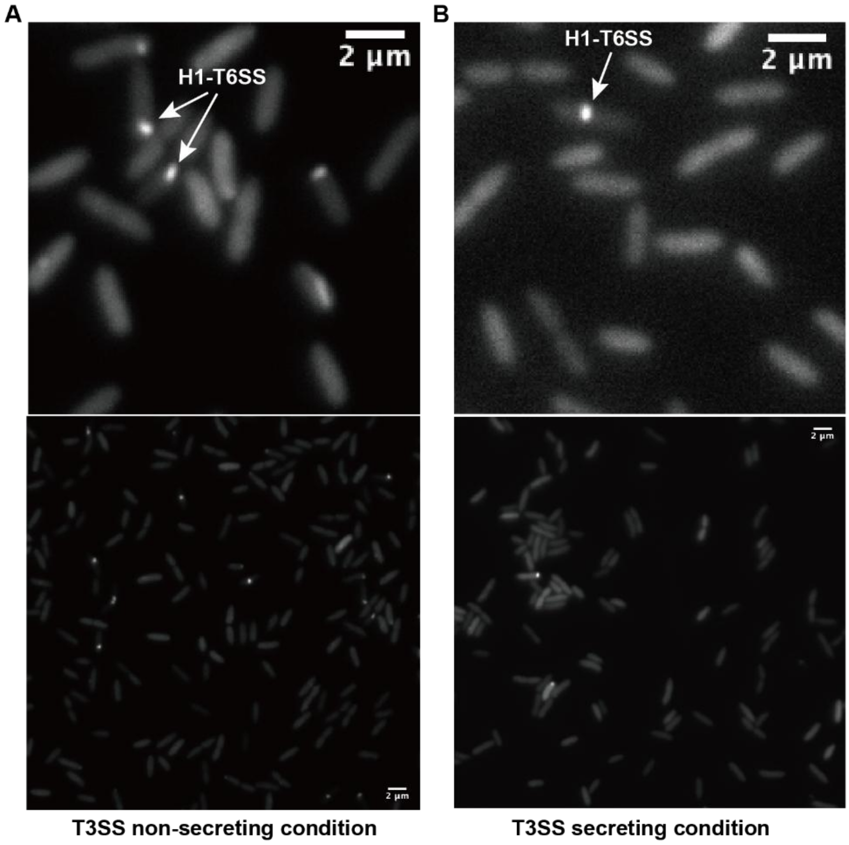

Suppl. Fig. 10 – Decreased H1-T6SS assembly in T3SS secreting conditions.

Representative microscopy pictures of H1-T6SS assembly in bacteria cultivated in T3SS non-secreting conditions (**A**) or secreting conditions (**B**). Top high magnification, bottom, larger field of view. Arrows indicate cells with assembled H1-T6SS.

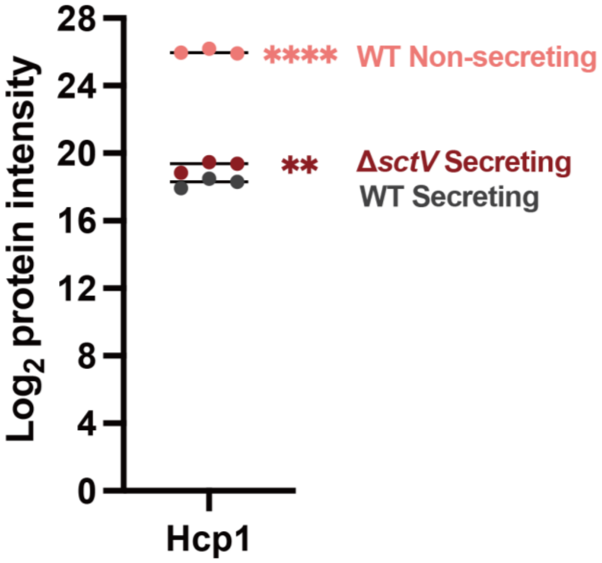

Suppl. Fig. 11 – Increased H1-T6SS secretion in T3SS-deficient strain and non-secreting conditions.

Label-free quantitative mass spectrometry analysis of the secretome of respective strains. WT and T3SS secretion deficient (Δ*sctV*) strains were cultivated in secreting and non-secreting conditions, as indicated. Data shows $\log_2$ of the signal intensity of the H1-T6SS effector Hcp1 in the indicated strains and conditions. Bars indicate the mean value. Biological replicates, n=3. Statistical analysis was done via ANOVA with multiple comparison to WT, **, $p< 0.01$; ****, $p< 0.0001$.

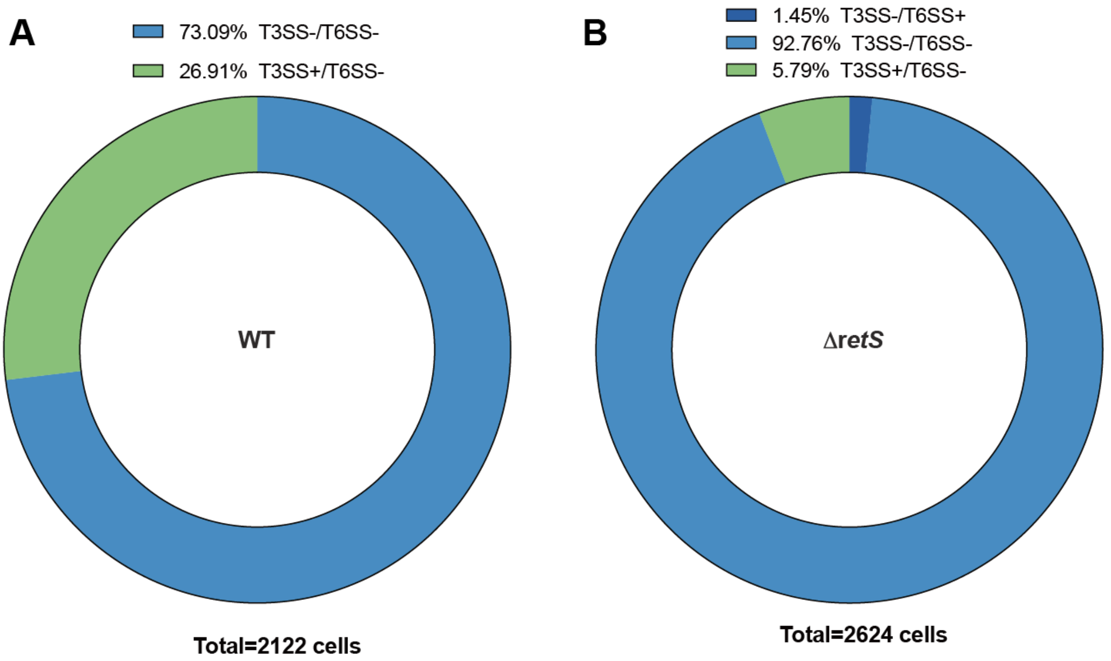

Suppl. Fig. 12 – Absence of RetS reduces the fraction of T3SS-positive bacteria, while increasing the fraction of T6SS-positive bacteria.

A) *P. aeruginosa* (EGFP-SctQ, TssB1-mCherry) were cultured in secreting conditions and tested for presence of the T3SS and the H1-T6SS. Data is shown as ratio of cells in specific status (H1-T6SS on/off and T3SS on/off) to total cells, exact ratio for each group is shown at the top, total cell amount tested is marked at the bottom. Samples were taken in 3 different experiments from 9 different fields with 100-200 cells each, total number of cells is indicated at the bottom. **B)** Same analysis in the corresponding Δ*retS* background strain. Samples were taken from 15 different fields in 3 different experiments.

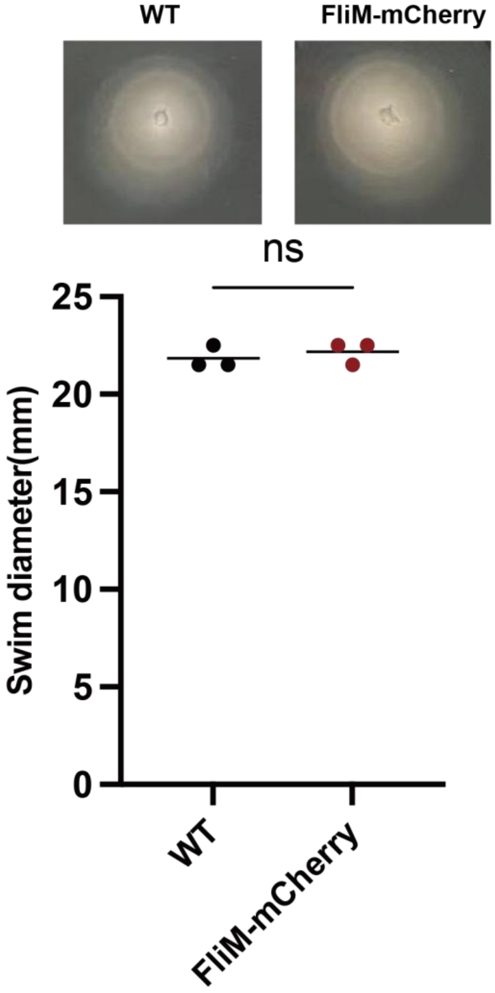

Suppl. Fig. 13 – FliM-mCherry fusion does not alter swimming ability.

The swimming diameter of WT and FliM-mCherry cultured in LB medium was tested. Swimming diameter was used as a proxy for motility because it provides an experimentally accessible, standardized metric, allowing for consistent comparison between different strains and experimental conditions. Representative swimming plates are shown at the top. Bars represent mean value. n=3 biological replicates. Statistical analysis was done via Student's *t* test; ns, non-significant.

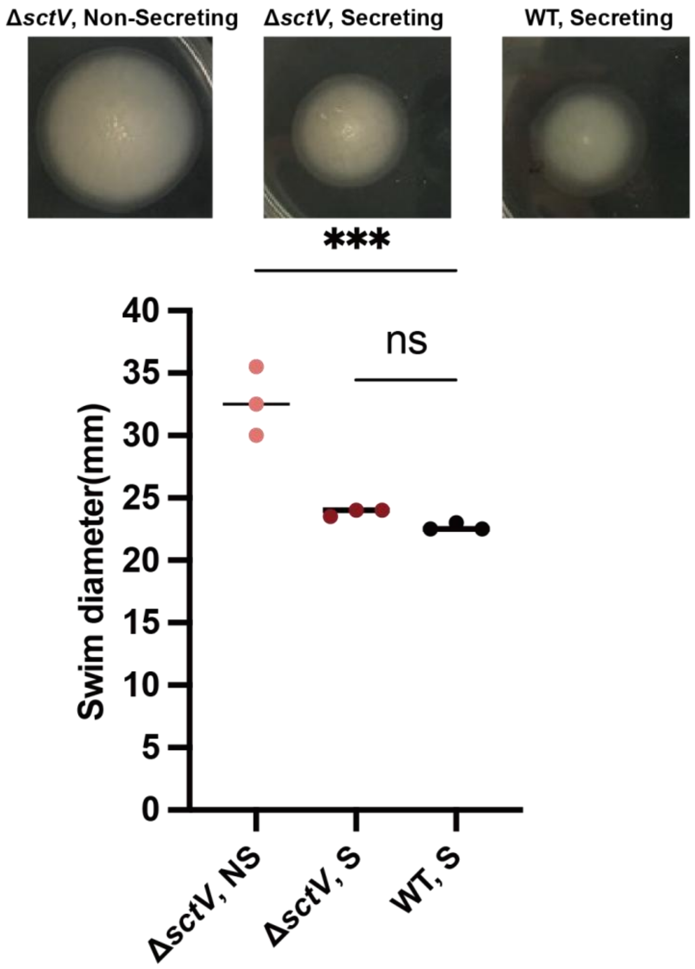

Suppl. Fig. 14 – Presence of the T3SS does not alter swimming ability.

The swimming diameter of T3SS secretion deficient (Δ*sctV*) and wild-type (WT) bacteria was tested in non-secreting conditions (abbreviated as NS) or secreting conditions (abbreviated as S). Representative swimming plates are shown at the top. Lines denote mean value. Biological replicates, n=3. Statistical analysis was done via ANOVA with multiple comparison to WT, ***, $p< 0.001$; ns, non-significant.

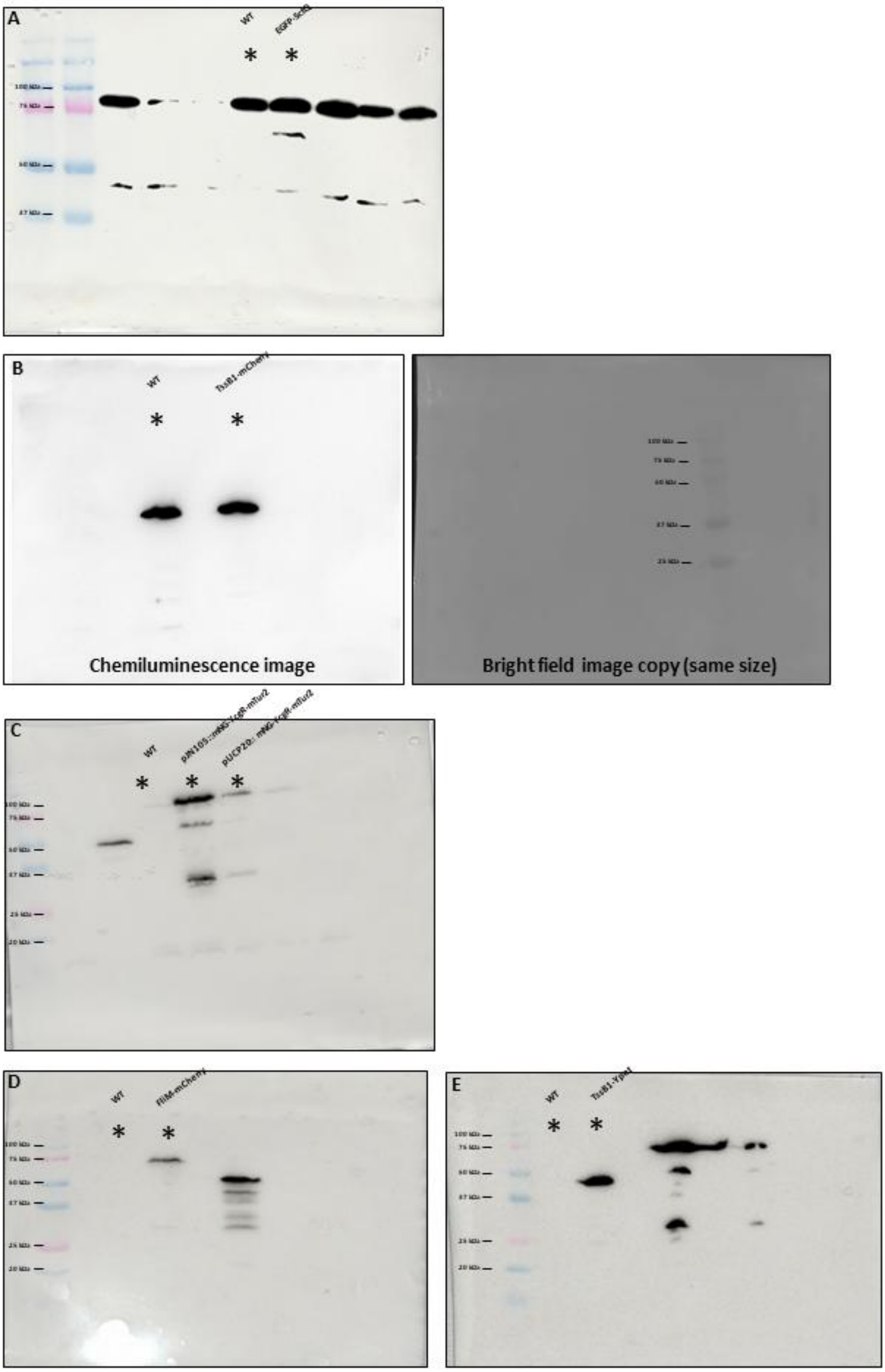

Suppl. Fig. 15 – Uncropped and unedited Western blot images.

Uncropped and unedited images of the Western blots shown in Suppl. Fig. 2, letters refer to panels in this figure. Overlays of light and luminescence images, except for B, where the two individual images are provided.

Suppl. Table. 1 – Nomenclature of key T3SS components.
Based on [1, 2].

Nomenclature		Function
Universal	*P. aeruginosa*	
**Cytosolic components**		
**SctQ**	PscQ	Cytosolic ring protein
**SctK**	PscK	Adaptor protein
**SctL**	PscL	Stator protein
**SctN**	PscN	ATPase
**SctO**	PscO	Stalk protein
**Export apparatus**		
**SctR**	PscR	Minor export apparatus protein
**SctS**	PscS	Minor export apparatus protein
**SctT**	PscT	Minor export apparatus protein
**SctU**	PscU	Export apparatus switch protein
**SctV**	PcrD	Major export apparatus protein (export gate)
**Basal body**		
**SctG**	ExsB	Pilotin
**SctC**	PscC	Outer membrane ring (secretin)
**SctD**	PscD	Inner membrane ring
**SctI**	PscI	Inner rod protein
**SctJ**	PscJ	Inner membrane ring
**Needle filament**		
**SctF**	PscF	Needle filament protein
**Translocation apparatus**		
**SctE**	PopB	Translocator protein
**SctB**	PopD	Translocator protein
**SctA**	PcrV	Needle-tip protein
**Regulation system**		
**SctW**	PopN	Gatekeeper
**SctP**	PscP	Needle length regulator

Suppl. Table. 2 - Strains and plasmids used in this study.

Gm^r, Str^r, Amp^r indicate Gentamicin, Streptomycin and Ampicillin resistance, respectively.

Strain	Genotype	Reference
PAO1	Wild-type *P. aeruginosa*	DSM 22644 (German Collection of Microorganisms, DSMZ)
DL001	PAO1 *egfp-sctQ*	[3]
DL002	PAO1 Δ*sctV*	[4]
DUK83	PAO1 Δ*tse6tsi6* SYFP2	This study
PAHZ007	PAO1 *egfp-sctQ tssB1-mCherry*	This study
PAHZ041	PAO1 *tssB1-mCherry* Δ*retS*	This study
PAHZ043	PAO1 *tssB1-yPet fliM-mCherry* Δ*retS*	This study
PAHZ044	PAO1 *egfp-sctQ fliM-mCherry*	This study
PAHZ045	PAO1 *egfp-sctQ tssB1-mCherry* Δ*retS*	This study
PAHZ046	PAO1 *egfp-sctQ tssB1-mCherry* Δ*retS* Δ*sctW*	This study
K207	PAO1 Δ*H1-T6SS* (Δ*PA0077-0091*)	This study
K245	PAO1 Δ*tssB1*	This study
OE241	PAO1 Δ*retS*	This study
**Plasmid**	**Genotype**	**Reference**
pJN105	pBBR1-derived L-arabinose inducible expression vector; Gm^r	[5]
pKNG101	Suicide vector; SacB; Str^r	[6]
pUCP20	Expression vector; Amp^r	[7]
pMMB67EH	Expression vector; Amp^r	[8]
pHZ002	pKNG101-FliM-mCherry	This study
pHZ007	pKNG101-TssB1-Ypet	This study
pHZ008	pKNG101-TssB1-mCherry	This study
pAD732	pJN105::PA2133-FLAG	This study
pHZ020	pJN105::YhjH-FLAG	This study
pHS002	pJN105::DgcA-FLAG	This study
pHZ060	pJN105::P_{exoS}-mCherry	This study
C3	pTrc99A::mNeonGreen-YcgR-mTurquoise2	[9]
pHZ064C	pJN105::mNeonGreen-YcgR-mTurquoise2	This study
pHZ065C	pMMB67EH::mNeonGreen-YcgR-mTurquoise2	This study
pHZ069	pKNG101-Δ*sctW*	This study

Suppl. Table. 3 - Primers used in this study.

Primers	Sequence (5'-3')	Used for
AD1023	GACTGAATTCATGAAAATCTCAGGCGCCCG	pHS002
AD1024	GACTTCTAGATCAAGCGCTCCTGCGCTTG	pHS002
AD1042	TATAGGGCCCGGACGAGCGCTGGATCAAGG	pHZ002
AD1043	CAATTGCGTAAGATCTGCCACCAGACCCGCCCGAACCACCGCGCGAGCGCTCGACCG	pHZ002
AD1044	GGTGGTTCGGGCGGGTCTGGTGGCAGATCTTACGCAATTGTGACCGCTTCCCCCGGAGG	pHZ002
AD1045	TATATCTAGACTCCAGGCCGGAAATGGTCG	pHZ002
AD1397	GACTGGGCCCCGCGTGCAGATCGAGTACG	pHZ007, pHZ008
AD1398	ACTGAGATCTACCACCAGAGCCGCCCGACCCACCCTGCGGCTCGTCGTCTTTG	pHZ007, pHZ008
AD1399	GGCGGCTCTGGTGGTAGATCTCAGTGGTCTCCAATTGTAAGAGGATTCCAGCATGGCCG	pHZ007, pHZ008
AD1400	GACTTCTAGACTTCTTGAAGATCGGGCTCTGG	pHZ007, pHZ008
AD1206	TATAGAATTCATGAACGGTTCCCCACAGG	pAD732
AD1207	TATATCTAGATCACTTATCATCGTCGTCCTTGTAGTCACCTCCTTGTCTGCTCGCCAGCGCCTCGA	pAD732
AD1457	GACTGAATTCATGATAAGGCAGGTTATCCAGCG	pHZ020
AD1458	CGATTCTAGATCACTTATCATCGTCGTCCTTGTAGTCACCTAGCGCCAGAACCGCC	pHZ020
AD1921	AGCTTCTAGAGGCAGCCATTAGAGCAGTGC	pHZ060
AD1923	GGATCCTTACTTGTACAGCTCGTCCATGCC	pHZ060
AD1992	CTAGGAATTCTGAAGGAGTGTGCCATGGGT	pHZ064C
AD1993	ACTGCTGCAGTCAGACCGCTTCTGCGTTC	pHZ064C
AD1994	CTAGCTGCAGCTGTTGACAATTAATCATCCGGCTCG	pHZ065C
AD1995	ACTGGAATTCTCAGACCGCTTCTGCGTTC	pHZ065C
AD2142	GACTGGATCCTTGGAGGAAACCCCGAGCAT	pHZ069
AD2143	CCGTATGCCATTGTGGTTCCTGGTCTGCAAAGG	pHZ069
AD2144	AGACCAGGAACCACAATGGCATACGGGCCTTCTGAA	pHZ069
AD2145	ATCGGGGCCCCAGCCAGAGCGACAACTGTTC	pHZ069
retS-A	ATA TAA GGT ACC ACG AAG CCG AAG CCG CCG	OE241
retS-B	GCCCTCAGGAGGGCAGGGCCCGAAGCCGTACCACGGCG	OE241
retS-C	CGCCGTGGTACGGCTTCGG GCCCTGCCCTCCTGAGGGC	OE241
retS-D	CTAAGTAAGCTTTGAGAGGAATGGTCAGCGGG	OE241
H1-T6SS-A	AAGCTTGGACGTAGCTGTGGTCCCGCGAGATGGC	K207
H1-T6SS-B	CGCTGGCGTAGAGGTTGAACTGCGGCTGCGCATCGACG	K207
H1-T6SS-C	CGTCGATGCGCAGCCGCAGTTCAACCTCTACGCCAGCG	K207
H1-T6SS-D	GGTACCCTACCTGAACAACAAGTTCGGTCGTTCCGGCAA	K207
tse6tsi6-A	GAATTCCGCCACGACCAGTCGAACGAGAACAGCTCC	DUK83
tse6tsi6-B	GACGCATGGATGCGCAAGCCGTCGATCTGCCCTGAGCGC	DUK83
tse6tsi6-C	GCGCTCAGGGCAGATCGAC GGCTTGCGCATCCATGCGTC	DUK83
tse6tsi6-D	TCTAGA CGAGATCAGTTCCAGCTCGTAGGCGAACG	DUK83
tssB1-A	AAGCTT GAC GGC GTC TAT CCG TTG CTC GAC G	K245
tssB1-B	CCTCTTACGCCTGCGGCTCGGTAGTGCTTCCCATCTTG	K245
tssB1-C	CAAGATGGGAAGCACTACC GAGCCGCAGGCGTAAGAGG	K245
tssB1-D	GGTACC GTCGGTTTCTTCCTCGAAGGCGAACTCTTCCAC	K245

Suppl. Table. 4 - LCMS/MS parameter for c-di-GMP measurements.

Name	Precursor Ion	Product Ion	Collision energy [V]	Fragmentor Voltage [V]	Cell Accelerator Voltage [V]	Dwell time [msec]	Polarity
Cyclic-di-GMP (Quantifier)	689.1	344	37	380	5	150	negative
Cyclic-di-GMP (Qualifier)	689.1	689.1	0	380	5	150	negative

References

[1] Hueck, C.J. (1998) 'Type III Protein Secretion Systems in Bacterial Pathogens of Animals and Plants', *Microbiology and Molecular Biology Reviews*, 62(2), pp. 379–433. Available at: https://doi.org/10.1128/MMBR.62.2.379-433.1998.

[2] Wagner, S. and Diepold, A. (2020) 'A Unified Nomenclature for Injectisome-Type Type III Secretion Systems', in, pp. 1–10. Available at: https://doi.org/10.1007/82_2020_210.

[3] Wimmi, S. *et al.* (2021) 'Dynamic relocalization of cytosolic type III secretion system components prevents premature protein secretion at low external pH', *Nature Communications*, 12(1), p. 1625. Available at: https://doi.org/10.1038/s41467-021-21863-4.

[4] Lampaki, D., Diepold, A. and Glatter, T. (2020) 'A Serial Sample Processing Strategy with Improved Performance for in-Depth Quantitative Analysis of Type III Secretion Events in *Pseudomonas aeruginosa*', *Journal of Proteome Research*, 19(1), pp. 543–553. Available at: https://doi.org/10.1021/acs.jproteome.9b00628.

[5] Kaur, D., Singh, V. and Gupta, S. (2024) 'Optimizing CaCl2-mediated transformation of Pseudomonas aeruginosa SDK-6 with pJN105 using OFAT: A novel and efficient cloning approach', *Current Genetics*, 70(1), p. 11. Available at: https://doi.org/10.1007/s00294-024-01295-5.

[6] Kaniga, K., Delor, I. and Cornelis, G.R. (1991) 'A wide-host-range suicide vector for improving reverse genetics in Gram-negative bacteria: inactivation of the blaA gene of Yersinia enterocolitica', *Gene*, 109(1), pp. 137–141. Available at: https://doi.org/10.1016/0378-1119(91)90599-7.

[7] Bense, S. *et al.* (2019) 'Spatiotemporal control of FlgZ activity impacts *Pseudomonas aeruginosa* flagellar motility', *Molecular Microbiology*, 111(6), pp. 1544–1557. Available at: https://doi.org/10.1111/mmi.14236.

[8] Lee, M.D. and Henk, A.D. (1997) 'RSF1010-based shuttle vectors for cloning and expression in Pasteurella multocida', *Veterinary Microbiology*, 54(3–4), pp. 369–374. Available at: https://doi.org/10.1016/S0378-1135(96)01294-1.

[9] Wang, L. *et al.* (2024) 'A toolbox of FRET-based c-di-GMP biosensors and its FRET-To-Sort application for genome-wide mapping of the second messenger regulatory network'. Available at: https://doi.org/10.1101/2024.08.21.609041.
