# Supplementary material for: Coordination of virulence factors and lifestyle transition in Pseudomonas aeruginosa through single-cell analysis
Source: Commun Biol. 2025 Aug 16;8:1236. doi: 10.1038/s42003-025-08693-6 (PMC12357884; doi:10.1038/s42003-025-08693-6)
Supplement: Supplementary file 3 — Description of Additional Supplementary Files [file 42003_2025_8693_MOESM3_ESM.pdf]

Description of Additional Supplementary Files

File name: Supplementary Data 1

Description: all numerical data values and p values
